# Effects of Nitisinone on Oxidative and Inflammatory Markers in Alkaptonuria: Results from SONIA1 and SONIA2 Studies

**DOI:** 10.3390/cells11223668

**Published:** 2022-11-18

**Authors:** Daniela Braconi, Michela Geminiani, Eftychia Eirini Psarelli, Daniela Giustarini, Barbara Marzocchi, Ranieri Rossi, Giulia Bernardini, Ottavia Spiga, James A. Gallagher, Kim-Hanh Le Quan Sang, Jean-Baptiste Arnoux, Richard Imrich, Mohammed S. Al-Sbou, Matthew Gornall, Richard Jackson, Lakshminarayan R. Ranganath, Annalisa Santucci

**Affiliations:** 1 Department of Biotechnology, Chemistry and Pharmacy, University of Siena, 53100 Siena, Italy; 2 Liverpool Clinical Trials Centre, University of Liverpool, Liverpool L69 3BX, UK; 3 UOC Clinical Pathology, Siena University Hospital, 53100 Siena, Italy; 4 Department of Musculoskeletal Biology, University of Liverpool, Liverpool L69 3BX, UK; 5 Hôpital Necker-Enfants Malades, CEDEX 15, 75015 Paris, France; 6 Biomedical Research Center, Slovak Academy of Sciences, 845 05 Bratislava, Slovakia; 7 Department of Pharmacology, Alkaptonuria Research Office, Faculty of Medicine, Mutah University, Mutah, Karak 61710, Jordan; 8 College of Medicine, Ajman University, Ajman P.O. Box 346, United Arab Emirates; 9 Department of Clinical Biochemistry and Metabolism, Royal Liverpool University Hospital, Liverpool L7 8XP, UK

**Keywords:** amyloidosis, biomarkers, disease severity, inborn errors of metabolism, inflammation, oxidative stress, protein carbonyls, rare disease, serum amyloid A (SAA)

## Abstract

Nitisinone (NTBC) was recently approved to treat alkaptonuria (AKU), but there is no information on its impact on oxidative stress and inflammation, which are observed in AKU. Therefore, serum samples collected during the clinical studies SONIA1 (40 AKU patients) and SONIA2 (138 AKU patients) were tested for Serum Amyloid A (SAA), CRP and IL-8 by ELISA; Advanced Oxidation Protein Products (AOPP) by spectrophotometry; and protein carbonyls by Western blot. Our results show that NTBC had no significant effects on the tested markers except for a slight but statistically significant effect for NTBC, but not for the combination of time and NTBC, on SAA levels in SONIA2 patients. Notably, the majority of SONIA2 patients presented with SAA > 10 mg/L, and 30 patients in the control group (43.5%) and 40 patients (58.0%) in the NTBC-treated group showed persistently elevated SAA > 10 mg/L at each visit during SONIA2. Higher serum SAA correlated with lower quality of life and higher morbidity. Despite no quantitative differences in AOPP, the preliminary analysis of protein carbonyls highlighted patterns that deserve further investigation. Overall, our results suggest that NTBC cannot control the sub-clinical inflammation due to increased SAA observed in AKU, which is also a risk factor for developing secondary amyloidosis.

## 1. Introduction

Alkaptonuria (AKU) is a rare autosomal recessive metabolic disorder (MIM 203500) of phenylalanine and tyrosine catabolism causing considerable morbidity once early onset chronic osteoarthritis-like damage manifests around the third/fourth decade of life. This is due to increased circulating homogentisic acid (HGA, 2,5-dihydroxyphenylacetic acid) leading to the deposition of an ochronotic pigment onto connective tissues in the osteoarticular and cardiac compartments, which is associated with a variety of clinical manifestations [1].

The use of nitisinone (NTBC, Orfadin) was suggested in AKU because it can block the production of HGA by inhibiting the enzyme hydroxyphenylpyruvate dioxygenase (HPPD) (EC 1.13.11.27), which is upstream of the defective 1,2-homogentisate dioxygenase (HGD) found in AKU. Therefore, a 4-week dose-finding study (Suitability of Nitisinone in Alkaptonuria 1, SONIA1) [2], and a four-year, open-label, evaluator-blinded, multicentre, randomized, no-treatment controlled, parallel-group study (SONIA2) were carried out to test the efficacy and safety of NTBC in AKU [3]. Results from these studies led to the approval of the use of NTBC for the treatment of AKU by the European Commission [4].

HGD mutations have no direct effect on the variability of the AKU phenotype, and relevant intra- and interindividual variability is found in urinary HGA levels [5]; therefore, other factors besides HGA should account for the variability observed in disease severity. There are so far no approved established clinical indicators that can describe AKU severity or predict disease prognosis. Hence, we undertook this work with a twofold aim: the evaluation of a potential short- or long-term effect of NTBC in modifying inflammatory and oxidative stress markers and the first longitudinal analysis of such markers in AKU in a 4-year timeframe.

## 2. Materials and Methods

### 2.1. Samples

Analyses were carried out in serum samples obtained from two clinical studies, namely SONIA1 (n = 40) and SONIA2 (n = 138). In SONIA1, patients were randomly allocated in five groups (n = 8): untreated (control) or treated with 1, 2, 4 or 8 mg NTBC for 4 weeks [2]. In SONIA2, patients were randomly allocated to two groups (n = 69): untreated (control) or treated with 10 mg NTBC for 4 years. A total of 55 patients in the NTBC group and 53 in the control group completed SONIA2 [3]. Serum samples were collected under fasting conditions and kept at −80 °C before analysis, as agreed within the research consortium. An independent Ethics Committee at each centre approved the study. Additional details on the study design are published elsewhere [2,3].

### 2.2. Measurements

In SONIA1 samples, serum SAA and interleukin 8 (IL-8) were measured using a magnetic bead-based multiplex assay (kit HCYTOMAG-60K, Milliplex, Millipore, Billerica, MA, USA) according to the manufacturer’s instructions. Plates were read on MagPix (Luminex DiaSorin Corporate, Saluggia (VC) Italy) using xPONENT Software (3.1 Rev2, Luminex DiaSorin Corporate, Saluggia (VC) Italy). Test analyte concentrations were obtained using a standard curve generated by Bio-Plex Manager software (version 6.1.1, Bio-Rad Laboratories S.r.l., Segrate (MI), Italy) with a 5-parameter logistic nonlinear curve-fitting method. C-reactive protein (CRP) was measured by means of commercial ELISA (catalog KHA0031, Invitrogen-Life Technologies, Camarillo, CA, USA) according to the manufacturer’s instructions. Plates were read on a VersaMax microplate reader (Molecular Devices, San Jose, CA, USA) using Ascent software (version 2.6, Thermo Scientific, Waltham, MA, USA). Quantification of analytes was obtained against polynomial standard curves generated with appropriate standards.

In SONIA2 samples, serum SAA was measured by means of commercial ELISA (catalog KHA0011, Invitrogen-Life Technologies, Camarillo, CA, USA) according to the manufacturer’s instructions. Plates were read on a VersaMax microplate reader (Molecular Devices, San Jose, CA, USA) using Ascent software (version 2.6, Thermo Scientific, Waltham, MA, USA). Quantification of analytes was obtained against polynomial standard curves generated with the appropriate standards.

AOPP in SONIA1 and SONIA2 samples was measured according to Witko-Sarsat et al. [6] by spectrophotometry on a microplate reader (Envision, Perkin Elmer, Milano, Italy) using Envision Manager software (version1.14, Perkin Elmer, Milano, Italy) as described previously [7].

For all the measurements, blanks, standards, and samples were tested at least in duplicate. Multiple samples from the same patient were always tested in the same plate. Measurements were performed at the end of study for SONIA1, whereas for SONIA2 an interim analysis was planned at year 1 together with a final analysis at year 4 after collection of all the samples. In this work, only the end of study results are reported (i.e., baseline and 1-year SONIA2 samples were re-tested, showing good reproducibility of results).

### 2.3. Western Blot of Protein Carbonyls

Selected SONIA2 serum samples were submitted to the analysis of protein carbonyls. Samples were first depleted of albumin and IgG with the Aurum Serum Protein Mini Kit (Bio-Rad Laboratories S.r.l., Segrate (MI), Italy), and the protein concentration was then assessed by Bradford’s assay. To derivatize protein carbonyls, ten micrograms of proteins were first incubated in the dark in 6% (*w*/*v*) sodium dodecyl sulphate (SDS), 5% (*v*/*v*) trifluoroacetic acid (TFA), and 5 mM 2,4-dinitrophenylhydrazine (DNPH); then the buffer reaction was neutralized with 2 M Tris base containing 30% (*v*/*v*) glycerol and 2% (*v*/*v*) β-mercaptoethanol [8]. Derivatized samples were submitted to SDS-PAGE (12% polyacrylamide), and gels were transferred to nitrocellulose (NC) sheets with the semidry Novablot transblot cell (Bio-Rad Laboratories S.r.l., Segrate (MI), Italy), applying 0.85 mA/cm^2^ for a total time of 75 min. For the detection of protein carbonyls, NC sheets were incubated overnight at 4 °C with 1:10,000 rabbit anti-dinitrophenyl antibodies (Sigma-Aldrich, Merk Life Science S.r.l., Milan, Italy), followed by 1:7000 HRP-conjugated anti-rabbit antibodies (Sigma-Aldrich) (2 h, room temperature) before enhanced chemiluminescence (Immobilon Crescendo Western HRP substrate, Merk Life Science S.r.l., Milan, Italy). In parallel, non-derivatized serum samples were run by SDS-PAGE (12% polyacrylamide) and gels were stained with Coomassie brilliant blue. A semiquantitative analysis was then undertaken on immunoreactive bands, whose volumes were subtracted of the background and normalized against the volumes of the corresponding bands in the replica Coomassie stained gel by calculating fold-changes values (end-of-study vs. baseline). Thresholds were set at 0.5 and 2.0 for significantly reduced or increased carbonylation, respectively.

### 2.4. Patients’ Data

SONIA2 patient’s age, clinical and quality of life data are reported in Table 1 according to information collected at investigative sites at yearly visits by using the following validated health questionnaires (additional information in Supplementary Table S1):

- Knee injury and Osteoarthritis Outcome Score (KOOS)
- Health Assessment Questionnaire (HAQ)
- Short Form-36 (SF-36)

Furthermore, specific items were extrapolated from a structured questionnaire named AKUSSI [9] that is specific to describe AKU: ear and eye ochronosis, patient joint and spinal pain, scintigraphy-based evaluation of osteoarticular disease of joints and spine, and overall clinical AKUSSI (cAKUSSI) score.

Serum HGA, serum tyrosine and 24-h urinary HGA were measured at Liverpool University Hospital [3].

### 2.5. Statistical Analysis

Raw data were stored in Excel (Microsoft 365, Microsoft corporation) and processed with GraphPad Prism 8 (version8.0.2, GraphPad Software Inc., San Diego, CA, USA); graphs were generated with GraphPad Prism 8 or Plotly [10] (Plotly Technologies Inc., Montréal, QC, Canada, available at https://plot.ly, accessed on 1 November 2022). Normal distribution was analyzed with a D’Agostino-Pearson test. Normally distributed data were expressed as mean ± standard deviation, and differences between groups were assessed using *t*-tests. Non- normally distributed data were expressed as median with 25 and 75 percentiles, and differences between groups were assessed using Mann–Whitney U-tests. A mixed-effects model analysis (REML) was carried out on log-transformed normalized data from repeated measurements to check for a statistically significant effect of treatment or the combination of time and treatment, followed by Dunnett’s or Sidak’s test for multiple comparisons. A Kruskal-Wallis’ test followed by Dunn’s multiple comparisons test was used to assess differences between groups, and Spearman’s test was used for correlation analyses. The statistical significance threshold was set at *p* ≤ 0.05.

## 3. Results

In this work, we investigated the effects of the drug NTBC on oxidative and inflammatory markers in alkaptonuric subjects after 4 weeks (SONIA1) and 4 years (SONIA2) of treatment. Untreated alkaptonuric subjects were in the control groups of both the studies. The measured markers were agreed upon within the research consortium and they were also based on previous results from our group [7].

### 3.1. SONIA1

The results obtained measuring IL-8, CRP, and SAA in SONIA1 are available in Supplementary Table S2. Since data were non normally distributed, they were log-transformed and analyzed by two-way repeated measures ANOVA corrected for multiple comparisons. We found that time, treatment with NTBC, and the combination of time and treatment had no significant effect on CRP (*p* = 0.0722, *p* = 0.729, and *p* = 0.5310, respectively) and SAA (*p* = 0.1039, *p* = 0.3065, and *p* = 0.6519, respectively). For IL-8, time (*p* = 0.569) and treatment (*p* = 0.3043) had no significant effect, whereas their interaction produced a statistically significant effect (*p* = 0.0194). A multiple comparison analysis (Table 2) showed an isolated a statistically significant decrease in IL-8 for patients treated with 4 mg NTBC/day compared to the untreated control (log mean control = 0.8711, log mean NTBC 4 mg = 0.3608, 95% CI [0.05383; 0.9669]) at the end of the study, which, however, is more likely due to the small size of groups or inter-individual variability, since a clear dose-response trend was missing (i.e., a higher NTBC dosage produced no statistically significant effect).

### 3.2. SONIA2

The results obtained measuring AOPP and SAA in SONIA2 are shown in Supplementary Table S3. Most of the patients either in the control or the NTBC-treated group presented with SAA levels above the reference threshold of 10 mg/L [11], with percentages spanning from 75.4% (control group, year 3) to 89.6% (NTBC-treated group, year 1). Notably, 30 patients (43.5%) in the control group and 40 patients (58.0%) in the NTBC-treated group showed persistently elevated SAA levels > 10 mg/L at each visit during the whole 4-year study (Supplementary Table S3).

**Figure 1:**
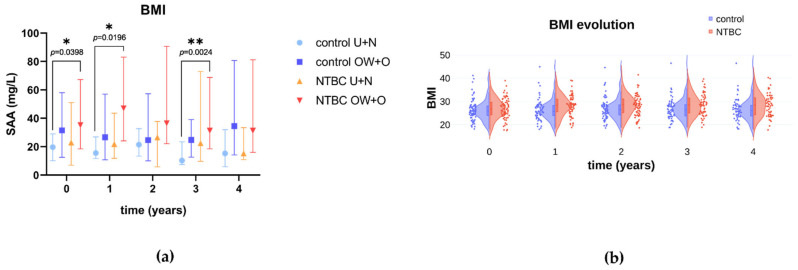
SAA and BMI in SONIA2. (**a**) SAA serum levels according to BMI in AKU subjects classified as: U + N, underweight and normal weight (BMI < 25); OW + O, overweight and obese (BMI ≥ 25). Median values are indicated with symbols, 25–75 percentiles with vertical lines. (**b**) Violin plot showing the evolution of BMI in control and NTBC. Dots indicate single values; internal boxes show median and 25–75 percentiles. A Kruskal-Wallis test followed by Dunn’s test for multiple comparisons was carried out. * *p* ≤ 0.05; ** *p* ≤ 0.01 (adjusted values).

Since data were non normally distributed, they were log-transformed and analyzed by a mixed effect repeated measures analysis. We found that neither the treatment with NTBC (*p* = 0.8173), nor the combination of time and treatment (*p* = 0.4860) had statistically significant effects on AOPP levels. Further analyses corrected for multiple comparisons showed no differences in AOPP values between the control and NTBC-group at each visit (Table 3). On the contrary, we found a slight but statistically significant effect for the treatment with NTBC (*p* = 0.022), but not for the combination of time and treatment (*p* = 0.2077), on SAA levels. Further analyses corrected for multiple comparisons showed that SAA levels were significantly higher in the NTBC-treated group compared to the untreated control at year 3 (log mean control = 1.265; log mean NTBC = 1.503; 95% CI [−0.4398; −0.03570]) (Table 3). However, a clear trend was lacking since SAA levels at year 4 did not show a statistically significant difference. Inter-individual variability along with the wide dynamic range of SAA might explain this isolated finding. Additionally, a multiple comparison analysis on SAA stratified by subjects’ BMI revealed that SAA was higher in NTBC-treated overweight/obese subjects compared to underweight/normal weight untreated subjects (Figure 1a). This phenomenon reached statistical significance at baseline, year 1 and year 3 (but the trend was observed along the entire study), and might contribute to the increase in SAA in the NTBC group. In fact, NTBC had a statistically significant effect on BMI (Table 1), whose evolution shows a clear increasing trend in the NTBC group compared to the control (Figure 1b).

**Figure 2:**
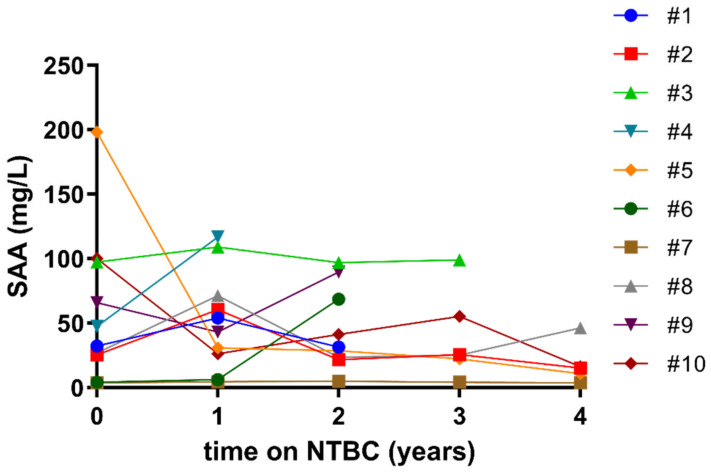
SAA serum levels in SONIA2 NTBC-treated subjects who developed keratopathy due to the NTBC-induced hypertyrosinemia. The graph shows the trend of serum SAA in these ten subjects for the period they could remain in the study.

Variability in SAA levels was observed in the small subset of NTBC-treated patients (n = 10) that developed keratopathy due to the NTBC-induced hypertyrosinemia (Figure 2): their serum SAA ranged from 4.2 mg/L (case #6, baseline) to 198.2 mg/L (case #5, baseline). In six patients out of ten, serum SAA remained stable (patients #1, #2, #3, #7, #8 and #9). For cases #5 and #10, who presented with high serum SAA at baseline (198 and 100 mg/L, respectively), sustained reduction in SAA was observed at the following visits, and both of the patients were able to complete the study. Conversely, for case #4, a >2-fold-increase was observed from baseline to year 1 before the patient’s dropout; similarly, for case #6 a 100-fold increase was observed from year 1 to year 2 before patient’s dropout.

**Figure 3:**
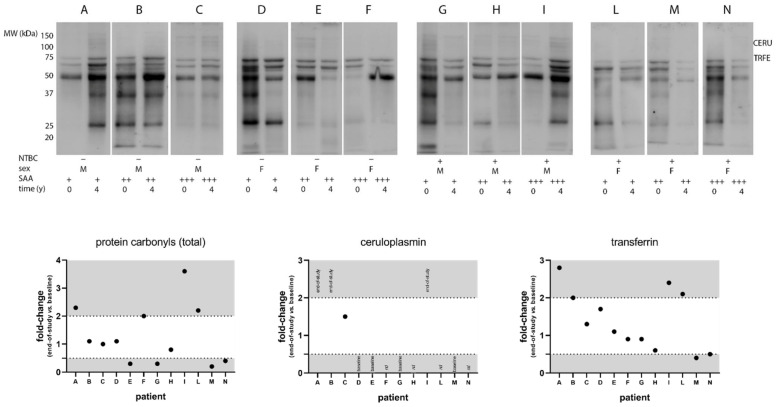
Analysis of protein carbonyls in a small group of SONIA2 patients’ sera. Baseline and end-of-study samples were selected according to sex, treatment, and SAA levels [low (+) if < 10 mg/L, medium (++) if between 10 and 50 mg/L, and high (+++) if > 50 mg/L]. Representative images from Western blot (upper panel) underwent a semi-quantitative analysis (lower panel) by calculating end-of-study vs. baseline fold-change values on normalized band volumes. Thresholds were set at 0.5 and 2 (dotted lines) for significantly reduced or increased carbonylation, respectively. Total carbonyls and specific proteins whose molecular weight is compatible with that of ceruloplasmin (CERU) and transferrin (TRFE) are reported. For CERU, the labels “end-of-study” and “baseline” indicate carbonylation only at these specific time points; *nd*: not detected. Additional details on these samples are available in Supplementary Table S4.

A preliminary analysis of protein carbonylation was carried out on a selected group of SONIA2 samples chosen to represent males and females (n = 6) equally, control and NTBC-treated subjects (n = 6), and SAA levels found to be low (<10 mg/L, n = 4), medium (10–50 mg/L, n = 4) or high (>50 mg/L, n = 4). Additional details on these samples can be found in Supplementary Table S4. To better visualize low abundant proteins, serum samples were previously depleted of albumin and immunoglobulins before being submitted to SDS-PAGE and Western blot. The semi-quantitative analysis suggested that despite no quantitative differences in AOPP levels in SONIA2 (Table 3), serum proteins could undergo quite variable carbonylation patterns (Figure 3). By looking at total carbonyls, increased levels were found in four cases (A, F, I, and L), reduced levels in four cases (E, G, M, and N) and no significant differences in the remaining four cases (B, C, D, and H) comparing end of study to baseline regardless of treatment or SAA levels (Supplementary Table S4). It was also interesting to note that specific protein bands whose apparent molecular weight was consistent with that of ceruloplasmin (≈115–135 kDa) and transferrin (≈75 kDa), as well as various protein bands that still need identification (data not shown), followed a similar trend. An increase in protein carbonylation was previously found when treating in vitro human serum with hypochlorous acid, which corroborates the finding that inflammation and protein oxidation are linked in several diseases [12]. Therefore, future investigations will be undertaken to shed light on this phenomenon and to evaluate if the oxidation of specific serum proteins can be used as an (early) biomarker of inflammatory conditions.

### 3.3. Correlation analysis in SONIA2

A correlation analysis carried out on end of study measurements showed that SAA was significantly correlated to patients’ BMI but not age (Table 4). Additionally, statistically significant correlations were found between SAA and patients’ reported outcomes on QoL (Table 4), as follows:

i. A positive correlation with the disability index of HAQ (haqdi), which describes the difficulty in performing some daily life activities; the higher the haqdi, the higher the difficulty.
ii. Negative correlations with the physical component of SF-36 (a lower score indicates higher disability), as well as activities of daily living, sport, and quality of life scores from the KOOS questionnaire (the lower the scores, the higher the knee-related problems).
iii. A positive correlation with the eye pigment score from cAKUSSI (the higher the score, the more abundant the ochronotic pigment).

No significant correlations were found for SAA and serum/urinary HGA or serum tyrosine levels (Table 4).

**Figure 4:**
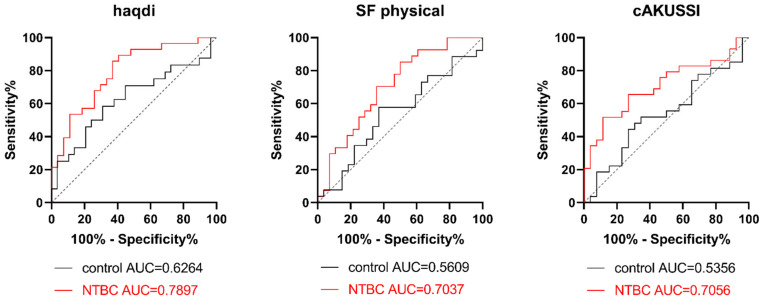
ROC curve of SAA for differentiating SONIA2 alkaptonuric subjects according to quality of life and disease severity in control and NTBC-treated groups. Abbreviations AUC: area under the curve; haqdi: Health Assessment Questionnaire disability index. SF physical: physical component of Short Form-36; cAKUSSI: clinical AKU Severity Score Index.

Based on these findings, SAA levels at the end of SONIA2 were generally correlated with lower QoL and higher morbidity in AKU. Furthermore, a ROC analysis showed that SAA could help differentiate SONIA2 patients according to the reported quality of life scores (haqdi and SF physical component) or disease severity (cAKUSSI), especially in the NTBC cohort (Figure 4).

## 4. Discussion

AKU represents an iconic inborn error of metabolism well characterized for its manifestations but whose molecular mechanisms are still incomplete [13,14,15,16,17,18]. Notably, AKU still lacks clinically relevant biomarkers to monitor severity and progression, and it was only recently that NTBC was approved for its treatment [4]. In such a framework, we undertook this work to monitor inflammation and oxidative stress biomarkers in AKU patients during treatment with NTBC.

Confirming previous reports [7,19,20,21,22,23,24] serum SAA levels but not AOPP were significantly increased in the vast majority of SONIA2 AKU patients regardless of treatment. In nearly half of them, SAA was persistently high throughout the study, which may be a risk factor for the development of AA amyloidosis [11], as observed in other rheumatological conditions [25,26,27,28]. Even younger AKU subjects showed significantly high SAA, which could help explain why cartilage deterioration, synovial inflammation, alterations in collagen composition, and proteoglycan depletion may be found in early asymptomatic stages [29]. In this work we also investigated for the first time the short- and long-term impact of NTBC on inflammatory and oxidative markers. If no effects were generally observed in the short term (SONIA1), in the long term (SONIA2) NTBC was associated with higher SAA. NTBC-treated patients were slightly older and with a more severe disease (i.e., higher cAKUSSI) at baseline (Table 1), which might represent potential confounding factors. More importantly, NTBC-treated patients gained weight due to a protein restricted diet to reduce the risk of keratopathy [30], which supports the positive correlation between SAA and BMI that we detected in SONIA2 patients at baseline [7] and confirmed here with end-of-study results. Obesity is linked to low-grade inflammation, the adipose tissue being a major source of SAA [31,32,33,34,35], and we have previously reported low HDL levels and altered lipoprotein profiles in AKU [7,20], which suggests the existence of a persistent inflammatory and amyloidogenic stimulus sustained by SAA similar to other rheumatological conditions [36,37,38,39]. We previously raised the possibility that HGA could sustain SAA production [7], but here we showed that SAA levels were persistently increased and pathologically relevant in vivo even when the HGA stimulus was removed by NTBC. Regardless, the cause of the SAA increase and the presence of potential confounding factors such as obesity or other concomitant inflammatory conditions, assessment and pharmacological control of SAA may be suggested in AKU [11,40,41,42,43]. This is also because HGA was found to promote the aggregation of SAA even at nearly physiological concentrations in vitro [44]; therefore, amyloid formation could represent an issue even for NTBC-treated subjects.

Despite the finding of normal AOPP ranges, our analysis showed that serum proteins in AKU can undergo variable carbonylation patterns. HGA and AKU were already linked to oxidation and carbonylation of serum proteins both in vitro and in vivo [20,45]. Here we showed that carbonylation might significantly affect important anti-oxidant serum proteins such as ceruloplasmin and transferrin [46,47,48], whose consequences deserve investigation.

Eye keratopathy is a major side effect of NTBC that seems to involve other factors than increased ocular tyrosine [49,50,51,52,53]. Inflammation and SAA-related pathways are associated to retinal microvascular changes [54] and pathological corneal neo-vascularization [55]. Furthermore, a role for SAA was suggested in the association between ankylosing spondylitis and acute anterior uveitis [56]. Since we highlighted a positive and significant correlation between SAA and eye ochronotic pigmentation here, we suggest that future research investigate whether SAA could concur with increased tyrosine to produce the ocular toxicity observed in AKU in NTBC-treated patients.

Though a systemic increase in HGA is observed in AKU, only tissues undergoing loading and stresses seem to be severely affected by ochronosis. Unfortunately, the measurement of SAA in the synovial fluid of SONIA2 patients was out of the scope of the trials, hence we can only speculate about the possibility of passive diffusion of SAA from blood to synovial joints, as already suggested by others [28,57,58]. Since both systemic and local SAA production can play a role in joint inflammation and destruction by matrix metalloproteinases [58], our findings suggest that persistently elevated SAA may represent a continuous stimulus promoting joint damage. This stress would be paralleled by HGA-induced mechanical and rheological alterations of cartilage and the presence of ochronotic pigment [16] co-localizing with amyloid deposits, further triggering local oxidative stress and inflammation [22,24,29,59,60].

An important limitation of SONIA2 was the fact that patients could not be blinded since NTBC, by blocking HGA production, prevents the typical urine discoloration which is pathognomonic of the disease [3]. Hence, bias could have affected patients’ reported outcomes about joint and spinal pain or QoL [61]. Other confounding factors are the presence of concomitant diseases contributing to morbidity, the use of medications other than NTBC (which were allowed during the study) to ease the pain, and prosthesis replacement that may have occurred during the study, all of them deserving further investigations. Nevertheless, as previously shown with SONIA2 baseline data [7], SAA was significantly correlated to various QoL and disease severity scores at the end of the study as well, with patients presenting with higher SAA reporting a lower QoL and higher morbidity. The ROC curve analysis presented here also suggests that SAA levels might be able to discriminate AKU patients based on disease severity. In line with previous reports in rheumatological conditions [28,62,63], our findings might thus support the use of serum SAA as a disease activity and severity marker in AKU. Similarly, it has been recently proposed that SAA could be used as a significant marker to track inflammation in COVID-19 infected patients, helping in the evaluation of disease severity and prognosis and allowing for personalization of treatment [64,65]. Although other biomarkers of inflammation have overwhelmed the use of SAA in the clinical practice, SAA has been demonstrated to provide more information and higher sensitivity, especially when subclinical inflammation is involved [64], emphasizing its clinical utility to monitor disease activity and treatment response.

## 5. Conclusions

To the best of our knowledge, this work analyzes for the first time the effects of the drug NTBC on biomarkers related to inflammation, oxidative stress, and amyloidosis in a relatively large cohort of subjects with the rare disease AKU. It also provides a longitudinal analysis of such markers in AKU, so far missing, as AKU patients were monitored at regular intervals for 4 years. The findings presented here suggest that: (i) NTBC cannot control the low grade, sub-clinical, chronic inflammation found in AKU due to increased SAA; and (ii) serum SAA should be monitored in AKU, as it represents a risk factor for the development of secondary amyloidosis and may promote local damage. Furthermore, our results might support the use of SAA as a clinically relevant disease activity and severity marker in AKU. Overall, the datasets generated here along with those already stored in our AKU database [66,67,68] could be amenable to further analyses through artificial intelligence tools [66,67,68,69,70,71], helping us to better understand AKU and the analysis of genotype-phenotype correlations, possibly leading to a precision-medicine approach to AKU.

## Figures and Tables

**Table 1:** SONIA2 patients’ demographic, clinical and quality of life data.

		Baseline			Year 4		
Variable		Control	NTBC	P ^§^	Control	NTBC	P ^§^
n		69	69		58	55	
age		47.59 ± 10.10	49.10 ± 11.39	0.4121	50.91 ± 9.778	52.07 ± 10.99	0.5554
BMI		26.00 (24.00, 28.35)	26.80 (24.25, 29.75)	0.3093	**26.10** **(23.70, 28.43)**	**28.50** **(24.25, 31.60)**	**0.0275**
HAQ	*visual analog scale*	48.00 (24.25, 70.75)	48.00 (25.00, 70.75)	0.6777	52.00 (27.00, 72.00)	49.00 (26.00, 70.00)	0.7214
	*disability* *index*	0.9348 ± 0.6480	0.8739 ± 0.6434	0.8739	0.880 (0.500, 1.380)	1.130 (0.630, 1.500)	0.2770
SF-36	*physical*	35.60 ± 10.87	35.46 ± 9.237	0.9349	35.71 ± 10.38	35.33 ± 9.715	0.8442
	*mental*	49.72 (38.12, 55.89)	49.72 (38.12, 55.89)	0.2064	42.00 (34.73, 52.49)	44.52 (37.02, 57.27)	0.5722
KOOS	*pain*	66.67 (47.22, 84.03)	65.28 (47.22, 90.98)	0.8965	66.67 (47.92, 90.98)	59.38 (50.00, 83.33)	0.6242
	*symptoms*	69.65 (49.11, 89.29)	69.65 (50.00, 89.29)	0.9336	75.00 (4732, 88.40)	67.86 (53.57, 85.71)	0.9715
	*activities of daily living*	66.18 (51.47, 91.55)	60.29 (42.28, 93.02)	0.5717	59.79 (45.96, 90.81)	57.35 (42.65, 80.88)	0.4988
	*sport*	45.00 (16.25, 80.00)	45.00 (10.00, 78.75)	0.7845	40.00 (16.25, 80.00)	40.00 (15.00, 71.25)	0.6486
	*quality of life*	53.13 (25.00, 81.25)	50.00 (18.75, 75.00)	0.7258	50.00 (25.00, 81.25)	43.75 (25.00, 75.00)	0.4206
cAKUSSI		82.00 (57.50, 105.0)	91.00 (56.00, 113.0)	0.2285	95.00 (69.25, 122.5)	97.00 (63.50, 123.8)	0.9354
sHGA (µmol/L)		27.30 (22.45, 32.90)	28.00 (22.45, 35.85)	0.4452	**33.10** **(24.28, 44.30)**	**0.5100** **(0.3400, 1.385)**	**<0.0001**
uHGA (µmol/L)		33,742 (26,102, 44,220)	33,291 (26,148, 42,767)	0.8023	**31,689** **(28,244, 38,260)**	**111.0** **(66.00, 312.0)**	**<0.0001**
sTyr (µmol/L)		64.52 ± 15.46	65.35 ± 15.46	0.7490	**61.50** **(54.75, 69.00)**	**813** **(692, 1013)**	**<0.0001**

Data expressed as mean ± SD (normally distributed variables) or median (25, 75 percentiles) (non-normally distributed variables). ^§^ P calculated with unpaired *t*-test (normally distributed variables) or Mann-Whitney test (non-normally distributed variables). Significant differences highlighted in bold. Abbreviations. HAQ: Health Assessment Questionnaire; SF-36: Short Form-36; KOOS: Knee injury and Osteoarthritis Outcome Score; cAKUSSI: clinical AKU Severity Score Index; sHGA: serum HGA; uHGA: urinary HGA; sTyr: serum tyrosine.

**Table 2:** Analysis of the effect of NTBC on inflammatory markers measured in SONIA1. Two-way repeated measures ANOVA corrected for multiple comparisons (Dunnett’s test, adjusted *p* values) was carried out on log transformed, normalized data. Statistically significant values are highlighted in bold.

		Baseline			Week 4		
		Mean	Mean Diff.	95% CI	Mean	Mean Diff.	95% CI
log IL-8 (pg/mL)	control	0.7384			0.8711		
	NTBC 1 mg	0.8264	−0.08796	[−0.5445; 0.3686]	0.7995	0.07169	[−0.3849; 0.5282]
	NTBC 2 mg	0.7413	−0.002917	[−0.4595; 0.4536]	0.6696	0.2015	[−0.2550; 0.6581]
	NTBC 4 mg	0.6581	0.08028	[−0.3763; 0.5368]	**0.3608**	**0.5104**	**[0.05383; 0.9669]**
	NTBC 8 mg	0.9006	−0.1622	[−0.6188; 0.2943]	0.7926	0.07851	[−0.3780; 0.5351]
log CRP (mg/L)	control	0.07317			0.2461		
	NTBC 1 mg	0.1300	−0.05686	[−0.6992; 0.5855]	0.3718	−0.1257	[−0.7680; 0.5166]
	NTBC 2 mg	0.1834	−0.1102	[−0.7525; 0.5322]	0.2459	0.0001408	[−0.6422; 0.6425]
	NTBC 4 mg	−0.1318	0.2049	[−0.4374; 0.8473]	0.09233	0.1538	[−0.4886; 0.7961]
	NTBC 8 mg	0.3177	−0.2445	[−0.8869; 0.3978]	0.2368	0.009251	[−0.6331; 0.6516]
log SAA (mg/L)	control	0.7569			0.7030		
	NTBC 1 mg	0.8183	−0.06140	[−0.5853; 0.4625]	1.007	−0.3045	[−0.8284; 0.2194]
	NTBC 2 mg	1.014	−0.2569	[−0.7808; 0.2669]	1.083	−0.3801	[−0.9040; 0.1437]
	NTBC 4 mg	0.7530	0.003929	[−0.5199; 0.5278]	0.8957	−0.1927	[−0.7166; 0.3312]
	NTBC 8 mg	1.053	−0.2958	[−0.8197; 0.2281]	1.141	−0.4383	[−0.9622; 0.08554]

**Table 3:** Analysis of the effect of NTBC on markers measured in SONIA2. A mixed-effects model and Sidak’s multiple comparisons test (adjusted *p* value) were carried out on log transformed, normalized data. Statistically significant value(s) are highlighted in bold.

Analyte	Time (Years)	Mean (Control)	Mean (NTBC)	Mean Diff.	95% CI
AOPP	0	1.026	1.014	0.01177	[−0.1136; 0.1372]
	1	1.024	0.9941	0.03017	[−0.09484; 0.1552]
	2	0.9165	0.945	−0.02851	[−0.1424; 0.08537]
	3	0.9097	0.9251	−0.0154	[−0.1229; 0.09211]
	4	0.9653	0.9704	−0.005068	[−0.1251; 0.1149]
SAA	0	1.367	1.448	−0.08166	[−0.2986; 0.1263]
	1	1.346	1.533	−0.1871	[−0.4007; 0.02657]
	2	1.369	1.533	−0.1644	[−0.3764; 0.04761]
	**3**	**1.265**	**1.503**	**−0.2378**	**[−0.4398; −0.03570]**
	4	1.389	1.451	−0.06242	[−0.3035; 0.1786]

**Table 4:** Correlation matrix for serum SAA in SONIA 2 patients at the end of study (year 4). Spearman’s r and *p* values are reported, and statistically significant correlations highlighted in bold. * *p* ≤ 0.05; ** *p* ≤ 0.01; *** *p* ≤ 0.001.

Source	Indicator	r	P
	age	−0.0278	ns
	BMI	0.369	*** <0.0001
HAQ	*hapvas*	0.136	ns
	*haqdi*	**0.360**	*** 0.0001
SF-36	*physical*	**−0.283**	** 0.0030
	*mental*	0.0320	ns
KOSS	*pain*	−0.170	ns
	*symptoms*	−0.180	ns
	*ADL*	**−0.247**	* 0.0102
	*sport*	**−0.231**	* 0.0175
	*QoL*	**−0.227**	* 0.0188
AKUSSI	*joint pain*	0.0445	ns
	*spinal pain*	−0.089	ns
	*eye pigment*	**0.309**	** 0.0012
	*ear pigment*	0.072	ns
	*ostearticular disease joints*	0.194	ns
	*ostearticular disease spine*	0.101	ns
	*cAKUSSI*	0.181	ns
metabolites	*sHGA*	−0.061	ns
	*u24HGA*	−0.078	ns
	*sTyr*	0.141	ns

Abbreviations: ns: not significant; hapvas: HA visual analog scale; haqdi: HA disability index; ADL: activities of daily living; QoL: quality of life. sHGA: serum HGA; u24HGA: 24 h urinary HGA; sTyr: serum Tyrosine.

## Data Availability

The data presented in this study are available on reasonable and qualified research request from the corresponding author. Proposals should be directed to daniela.braconi@unisi.it. Data requestors will need to sign a data access agreement.

## Supplementary Materials

The following supporting information can be downloaded at: https://www.mdpi.com/article/10.3390/cells11223668/s1. Table S1. Health questionnaires for SONIA2 patients. Table S2: Summary of measurements in SONIA1 patients. Table S3. Summary of measurements in SONIA2 patients. Table S4. Summary of measurements in SONIA2 samples submitted to the analysis of protein carbonyls.
