# Supplementary material for: Effects of Nitisinone on Oxidative and Inflammatory Markers in Alkaptonuria: Results from SONIA1 and SONIA2 Studies
Source: Cells. 2022 Nov 18;11(22):3668. doi: 10.3390/cells11223668 (PMC9688277; doi:10.3390/cells11223668)
Supplement: Supplementary file 1 [file cells-11-03668-s001.zip › cells-2040369-Supplementary.pdf]

Effects of nitisinone on oxidative and inflammatory markers in alkaptonuria: results from SONIA1 and SONIA2 studies

Supplementary material

Table S1. Health questionnaires for SONIA2 patients.

Questionnaire	Description and items	Scale
**Knee injury and Osteoarthritis Outcome Score (KOOS)**	Describes short- and long-term consequences of knee injury in five subscales [*pain, other symptoms, function in daily living, function in sport and recreation*, and *knee-related quality of life (QoL)*]	**0** (extreme problems)<br>**100** (no problems)
**Health Assessment Questionnaire (HAQ)**	Includes a *disability index (haqdi)* and a *global pain visual analog scale (hapvas)*	**0** (no difficulties)<br>**3** (unable to do).
**Short Form-36 (SF-36)**	A multi-purpose short form questionnaire with 36 questions addressing both *physical* and *mental* components	**0** (maximum disability)<br>**100** (no disability).

Table S2. Summary of measurements in SONIA1 patients.

		baseline		4 weeks	
		**n (min-max)**	**median (25-75 percentile)**	**n (min - max)**	**median (25-75 percentile)**
**IL-8 (pg/mL)**	control	8 (1.290 – 19.25)	6.080 (3.345 – 8.025)	8 (4.890 – 18.48)	5.905 (5.133 – 14.27)
	NTBC 1 mg	8 (2.290 – 14.27)	6.085 (5.338 – 12.03)	8 (3.950 – 11.42)	5.725 (4.593 – 9.048)
	NTBC 2 mg	8 (1.340 – 14.07)	8.670 (2.235 – 9.610)	8 (2.020 – 12.59)	4.930 (2.243 – 9.200)
	NTBC 4 mg	8 (0.480 – 17.65)	5.180 (2.950 – 9.060)	8 (0.060 – 12.47)	3.270 (2.120 – 5.198)
	NTBC 8 mg	8 (2.980 – 18.26)	9.505 (5.188 – 10.70)	8 (2.070 – 16.18)	5.810 (5.153 – 9.265)
**CRP (mg/L)**	control	8 (0.3253 – 8.032)	0.6508 (0.5052 – 5.223)	8 (0.2441 – 11.66)	1.263 (0.8081 – 7.048)
	NTBC 1 mg	8 (0.117 – 5.335)	1.990 (0.7422 – 3.009)	8 (0.1856 – 10.79)	2.490 (0.8890 – 8.628)
	NTBC 2 mg	8 (0.4564 – 20.83)	1.344 (0.5872 – 2.676)	8 (0.3774 – 16.30)	1.457 (1.103 – 3.103)
	NTBC 4 mg	8 (0.2243 – 2.016)	0.9551 (0.2721 – 1.788)	8 (0.3491 – 4.919)	1.612 (0.4391 – 2.232)
	NTBC 8 mg	8 (0.3728 – 8.275)	2.759 (0.5618 – 5.999)	8 (0.3925 – 7.709)	1.741 (0.6345 – 5.213)
**SAA (mg/L)**	control	8 (1.740 – 26.29)	5.104 (2.145 – 18.94)	8 (1.986 – 38.25)	3.109 (2.073 – 24.47)
	NTBC 1 mg	8 (1.527 – 21.89)	7.134 (2.911 – 15.62)	8 (2.601 – 57.40)	9.212 (4.036 – 26.75)
	NTBC 2 mg	8 (3.143 – 101.8)	9.510 (3.475 – 21.30)	8 (3.476 – 45.96)	14.73 (5.527 – 21.36)
	NTBC 4 mg	8 (2.476 – 16.61)	5.096 (3.045 – 13.80)	8 (2.029 – 37.26)	6.492 (6.293 – 12.69)
	NTBC 8 mg	8 (3.815 – 28.47)	12.84 (5.018 – 23.30)	8 (4.134 – 43.44)	14.95 (5.802 – 33.36)

Table S3. Summary of measurements in SONIA2 patients.

group	time (years)	AOPP µmol/dL (chlor T eq) n (min-max)	AOPP median (25-75 percentile)	AOPP n > 30 µmol/dL (%)	SAA (mg/L) n (min-max)	SAA median (25-75 percentile)	SAA n > 10 mg/L (%)
control	0	68 (2.06 – 58.84)	10.01 (7.72 – 13.92)	4 (5.9%)	69 (1.921 – 305.7)	26.21 (11.42 – 51.44)	54 (78.3%)
	1	63 (1.95 – 58.89)	10.13 (7.34 – 17.16)	4 (6.3%)	63 (1.951 – 228.2)	22.23 (10.89 – 53.15)	49 (77.8%)
	2	62 (1.81 – 45.49)	8.422 (6.24 – 10.90)	1 (1.6%)	61 (2.478 – 280.2)	22.25 (11.46 – 44.35)	47 (77.0%)
	3	61 (2.80 – 32.16)	8.131 (5.40 – 10.72)	1 (1.6%)	61 (2.386 – 202.2)	18.65 (9.969 – 34.18)	46 (75.4%)
	4	53 (1.85 – 39.18)	9.032 (6.90 – 12.02)	2 (3.8%)	54 (1.093 – 237.1)	29.16 (11.81 – 48.80)	43 (79.6%)
NTBC	0	69 (2.93 – 15.75)	10.56 (6.61 – 15.75)	3 (4.3%)	69 (0.788 – 311.9)	29.16 (15.34 – 64.55)	56 (81.2%)
	1	67 (2.34 – 30.0)	10.35 (6.66 – 14.10)	1 (1.5%)	67 (1.934 – 197.6)	41.28 (19.65 – 73.72)	60 (89.6%)
	2	65 (2.53 – 41.05)	8.869 (5.93 – 13.05)	2 (3.1%)	65 (0.765 – 260.8)	34.45 (21.11 – 86.33)	56 (86.2%)
	3	60 (1.43 – 20.82)	8.611 (6.31 – 12.76)	0	60 (3.011 -287.9)	31.07 (16.07 – 69.89)	51 (85.0%)
	4	56 (1.99 – 26.98)	8.675 (6.94 – 14.98)	0	56 (3.899 – 244.6)	27.52 (13.54 – 67.13)	47 (83.9%)

Table S4. Summary of measurements in SONIA2 samples submitted to the analysis of protein carbonyls. Significantly different carbonylation is indicated in bold: reduced if fold-change ≤0.5; increased if fold-change ≥ 2.

patient ID	arm	sex	SAA (mg/L) baseline	SAA (mg/L) end of study	AOPP µmol/dL (chlor T eq) baseline	AOPP end of study	cAKUSSI baseline	cAKUSSI end of study	fold-change in protein carbonylation (end-of-study *vs.* baseline) total	CERU	TRFE
A	control	M	1.921	1.931	3.469	3.754	138	166	**2.3**	carbonylated at end of study	**2.8**
B	control	M	20.123	28.232	11.438	11.954	58	53	1.1	carbonylated at end of study	**2.0**
C	control	M	94.077	158.257	13.518	12.084	66	85	1.0	1.5	1.3
D	control	F	6.412	8.486	10.887	6.878	42	47	1.1	carbonylated at baseline	1.7
E	control	F	28.561	17.187	17.162	10.567	65	93	**0.3**	carbonylated at baseline	1.1
F	control	F	110.118	133.575	9.288	10.199	106	115	**2.0**	not detected	0.9
G	NTBC	M	3.899	3.899	9.503	6.646	30	28	**0.3**	carbonylated at baseline	0.9
H	NTBC	M	29.159	29.895	7.102	7.350	106	120	0.8	not detected	0.6
I	NTBC	M	128.099	239.182	7.916	7.360	104	138	**3.6**	carbonylated at end of study	**2.4**
L	NTBC	F	3.802	6.152	4.278	1.987	85	92	**2.2**	not detected	**2.1**
M	NTBC	F	25.732	27.519	10.772	3.931	42	40	**0.2**	carbonylated at baseline	**0.4**
N	NTBC	F	126.888	99.182	5.819	7.087	94	101	**0.4**	not detected	**0.5**
